# The dendritic cell niche in chronic obstructive pulmonary disease

**DOI:** 10.1186/1465-9921-13-80

**Published:** 2012-09-19

**Authors:** Angela Haczku

**Affiliations:** 1 Pulmonary, Allergy and Critical Care Division, Translational Research Laboratories, 125 South 31st Street, Philadelphia, PA 19104-3403, USA

**Keywords:** COPD, Dendritic cells, IL-1R1, IL-1α, Cigarette smoke exposure, Mice

## Abstract

The pulmonary innate immune system is heavily implicated in the perpetual airway inflammation and impaired host defense characterizing Chronic Obstructive Pulmonary Disease (COPD). The airways of patients suffering from COPD are infiltrated by various immune and inflammatory cells including macrophages, neutrophils, T lymphocytes, and dendritic cells. While the role of macrophages, neutrophils and T lymphocytes is well characterized, the contribution of dendritic cells to COPD pathogenesis is still the subject of emerging research. A paper by Botelho and colleagues in the current issue of Respiratory Research investigates the importance of dendritic cell recruitment in cigarette-smoke induced acute and chronic inflammation in mice. Dendritic cells of the healthy lung parenchyma and airways perform an important sentinel function and regulate immune homeostasis. During inflammatory responses the function and migration pattern of these cells is dramatically altered but the underlying mechanisms are incompletely understood. Botelho and colleagues demonstrate here the importance of IL-1R1/IL-1α related mechanisms including CCL20 production in cigarette-smoke induced recruitment of dendritic cells and T cell activation in the mouse lung.

## Background

The average lungs filter through approximately 11,000 L air a day with numerous potentially toxic, infectious or allergenic particles landing on a football-field size respiratory surface area. Yet, the healthy respiratory mucosal surface is maintained in a pathogen and inflammation free state. The innate immune system has a constitutive daunting task of eliminating inhaled particles, educating the adaptive immune system (T cells and B cells) and preventing inappropriate inflammatory changes. This entails a tightly regulated cooperation between structural cells (fibroblasts, smooth muscles and epithelial cells), alveolar macrophages and dendritic cells. The latter normally reside in the lung parenchyma and airways, sampling the environment and regulating immune homeostasis in the proximal and distal air spaces. Acute inflammatory responses elicit an intricately organized hierarchy and trafficking of subpopulations of this highly heterogeneous cell type. The importance of these changes in COPD has been raised by a number of laboratories [1-6]. Many questions are however remain unanswered. For example, what are the danger signals and downstream pathways that induce dendritic cell changes in COPD? How do dendritic cell subpopulations differentiate and what is their significance in the chronically inflamed airways? What chemokine receptor-ligand pairs regulate accumulation of dendritic cells in the lung/airways? Is there a role for antigen presentation and the adaptive immune response in COPD? In the current issue of Respiratory Research Botelho and colleagues bring us closer to understanding some of these questions by assessing cigarette-smoke induced accumulation of dendritic cells in the lung in a relevant murine model [7].

### Danger signals for dendritic cell maturation and migration during the inflammatory airway response in COPD

Dendritic cells are professional antigen-presenting cells and regulators of immunity and tolerance. Under baseline conditions, these cells reside in the peripheral tissue in an immature, resting state scattered throughout the respiratory mucosal wall. They are capable of capturing pathogens but unable to present them to T cells in this state. In response to danger signals provided by pattern recognition receptors or proinflammatory cytokines, dendritic cells start to mature and switch from “antigen capturing” to “antigen presenting” and T-cell–stimulatory mode. To study the role of different danger signals in dendritic cell migration and activation during cigarette-smoke induced inflammatory responses, in the present paper Botelho and colleagues [7] investigated IL-1R1, TLR4 and IL-1α deficient mice as well as anti IL-1α and IL-1β blocking antibodies. Their results showed that accumulation and activation of dendritic cells were IL-1R1/IL-1α dependent but TLR4 and IL-1β independent. While both IL-1α [8]and IL-1β [9] are found significantly increased in COPD, the data presented here suggest that IL-1α specific pathways are important and that activation of inflammasome related events may not be required for dendritic cell maturation/migration. Given however that the NLRP3 and the TLR4/myD88 pathways are thought to be essential in cigarette smoke induced pulmonary inflammation in mice [10] it would be important to clarify whether these pathways are indeed redundant in regulating dendritic cell migration in COPD.

### Significance of dendritic cell subclasses

Dendritic cells originate from the monocyte and dendritic cell progenitor. Committed dendritic progenitors in the bone marrow give rise to pre-dendritic cells, which migrate from the bone marrow to lymphoid and non-lymphoid tissues where their maturation and differentiation is regulated by different cytokines and growth factors. Phenotypically different populations of conventional dendritic cells have been identified in the respiratory mucosal tissue. It is important to differentiate between dendritic cell subsets because they play very different roles such as inducing tolerance versus driving the immune response. According to cell surface marker profile, in the lung for example, the majority of the resident dendritic cells under baseline conditions are made up by the plasmocytoid type (120G8^high^/PDCA-1^high^/Gr1^high^/B220^high^) shown to be tolerogenic in allergen-induced inflammation. On the other hand, myeloid dendritic cells (CD11c^high^/CD11b^high^/MHC-II^high^) migrate rapidly to the lung during inflammation. The integrin CD103 (alpha(E)) was shown to denote a population of dendritic cells in skin, lung, and intestine that can efficiently present exogenous antigens in their corresponding draining lymph nodes to specific CD8+ T cells through cross-presentation. CD103+ dendritic cells also contribute to the control of inflammatory responses and mucosal homeostasis by fostering the conversion of naive T cells into induced Foxp3+ regulatory T cells, mediated by transforming growth factor-beta and retinoic acid signaling. Unfortunately, to date there is an almost complete absence of knowledge on how dendritic cell subsets are regulated in COPD. Although the paper by Bothelo et al. did not study any particular subclasses they demonstrated their findings on relevant CD11c^high^/MHC-II^high^/CD86^high^ cells that belong to the highly mature dendritic cell population with antigen presenting and CD4+ T cell stimulatory capabilities.

### Role of chemokines in dendritic cell accumulation in the lung in COPD

In addition to differential maturation, distinct patterns of tissue and lymph node directed migration determine dendritic cell function and the outcome of inflammatory response. Dendritic cells and other members of the innate and adaptive immune system migrate to peripheral and lymphoid tissues during inflammation and utilize over 40 chemokines and 20 chemokine receptors in this process [11,12]. Cigarette smoke was recently shown to induce activation of the CCL19-CCR7, CXCL1-CXCR2, CCL2-CCR2/4 and CCL20-CCR6 pathways, pertinent to dendritic cell migration in mice [3,13-15] and COPD patients [6]. Previous work [15] showed that lack of CCR6 in gene deficient mice inhibited dendritic cell accumulation and airway inflammation in cigarette smoke exposed mice, highlighting the importance of the CCL20-CCR6 ligand-receptor pair. Botelho et al. used an elegant bone marrow reconstitution experiment that resulted in CCL20 and GM-CSF production in response to cigarette smoke in wild type but not IL-1R1−/− recipient mice. This suggested the importance of IL-1R1 expression on structural cells in production of CCL20 and the ensuing dendritic cell accumulation in the cigarette smoke exposed lung.

### Is there a role for antigen presentation in COPD?

Although all mature dendritic cells share a common ability to process and present antigen to naive T cells for the initiation of an immune response, they are highly heterogeneous in origin, migratory patterns, localization, cytokine/mediator production and cell-surface marker expression. This heterogeneity in turn is important in determining the outcome of the immune response (i.e. tolerance vs. activation). In murine models of COPD dendritic cells were suggested to play a role by activation of autoreactive T cells and induce emphysema in mice in an IL-17A dependent fashion [16]. Although the paper by Botelho et al. did not investigate whether antigen presentation was involved, they showed diminished activation of CD4 and CD8 T cells in the IL-1R1−/− (but not the TLR4−/−) mice. This is actually not surprising given that T cells require IL-1 for activation. The role of dendritic cells in this effect will therefore need further investigation.

## Conclusions

Bothelo and colleagues demonstrated here that acute and chronic cigarette smoke exposure induces dendritic cell migration into the lung in an IL-1R1 dependent manner. They show the importance of IL-1α as opposed to IL-1β or TLR4 in this process, and imply an IL-1R1 driven CCL20 pathway. They also show that T cell activation is diminished in the absence of IL-1R1 and IL-1 α. This study highlights the role of dendritic cells in the pathogenesis of COPD and contributes to the foundation on which the underlying regulatory mechanisms maybe unraveled.

## Abbreviations

BAL: Bronchoalveolar lavage; CCL: CC chemokine ligand; CCR: CC chemokine receptor; CD: Cluster of differentiation; CXCR: CXC chemokine receptor; ELISA: Enzyme linked immunosorbent assay; GM-CSF: Granulocyte macrophage colony stimulating factor; IL: Interleukin; IL-1R1: Interleukin 1 receptor 1; LPS: Lipopolysaccharide; PBS: Phosphate buffered saline; TLR4: Toll like receptor 4; WT: Wild type.

## Competing interests

The author declares that she has no competing interests.

## Authors’ contributions

AH wrote the editorial.

## References

[B1] Brusselle GG Joos GF Bracke KR New insights into the immunology of chronic obstructive pulmonary disease Lancet 2011 378 1015 1026 10.1016/S0140-6736(11)60988-4 21907865

[B2] Van Pottelberge GR Bracke KR Demedts IK De Rijck K Reinartz SM van Drunen CM Verleden GM Vermassen FE Joos GF Brusselle GG Selective accumulation of langerhans-type dendritic cells in small airways of patients with COPD Respir Res 2010 11 35 10.1186/1465-9921-11-35 20307269 PMC2858735

[B3] Demoor T Bracke KR Vermaelen KY Dupont L Joos GF Brusselle GG CCR7 modulates pulmonary and lymph node inflammatory responses in cigarette smoke-exposed mice J Immunol 2009 183 8186 8194 10.4049/jimmunol.0902015 19923454

[B4] Van Pottelberge GR Bracke KR Joos GF Brusselle GG The role of dendritic cells in the pathogenesis of COPD: liaison officers in the front line COPD 2009 6 284 290 10.1080/15412550903049124 19811388

[B5] Tsoumakidou M Demedts IK Brusselle GG Jeffery PK Dendritic cells in chronic obstructive pulmonary disease: new players in an old game Am J Respir Crit Care Med 2008 177 1180 1186 10.1164/rccm.200711-1727PP 18337593

[B6] Demedts IK Bracke KR Van Pottelberge G Testelmans D Verleden GM Vermassen FE Joos GF Brusselle GG Accumulation of dendritic cells and increased CCL20 levels in the airways of patients with chronic obstructive pulmonary disease Am J Respir Crit Care Med 2007 175 998 1005 10.1164/rccm.200608-1113OC 17332482

[B7] Bothelo FM Nikota JK Bauer CMT Morissette MC Iwakura Y Kolbeck R Finch D Humbles AA Stampfli MR Cigarette-smoke induced accumulation of lung dendritic cells is interleukin-1α dependent in mice Respir Res 2012 6 e28457 10.1186/1465-9921-13-81 PMC3519608 22992200

[B8] Botelho FM Bauer CM Finch D Nikota JK Zavitz CC Kelly A Lambert KN Piper S Foster ML Goldring JJ IL-1alpha/IL-1R1 expression in chronic obstructive pulmonary disease and mechanistic relevance to smoke-induced neutrophilia in mice PLoS One 2011 6 e28457 10.1371/journal.pone.0028457 22163019 PMC3232226

[B9] Chung KF Inflammatory mediators in chronic obstructive pulmonary disease Curr Drug Targets Inflamm Allergy 2005 4 619 625 10.2174/156801005774912806 17305518

[B10] Doz E Noulin N Boichot E Guenon I Fick L Le Bert M Lagente V Ryffel B Schnyder B Quesniaux VF Couillin I Cigarette smoke-induced pulmonary inflammation is TLR4/MyD88 and IL-1R1/MyD88 signaling dependent J Immunol 2008 180 1169 1178 18178857 10.4049/jimmunol.180.2.1169

[B11] Bisset LR Schmid-Grendelmeier P Chemokines and their receptors in the pathogenesis of allergic asthma: progress and perspective Curr Opin Pulm Med 2005 11 35 42 10.1097/01.mcp.0000144502.50149.e0 15591886

[B12] Penna G Vulcano M Roncari A Facchetti F Sozzani S Adorini L Cutting edge: differential chemokine production by myeloid and plasmacytoid dendritic cells J Immunol 2002 169 6673 6676 12471096 10.4049/jimmunol.169.12.6673

[B13] Demoor T Bracke KR Dupont LL Plantinga M Bondue B Roy MO Lannoy V Lambrecht BN Brusselle GG Joos GF The role of ChemR23 in the induction and resolution of cigarette smoke-induced inflammation J Immunol 2011 186 5457 5467 10.4049/jimmunol.1003862 21430224

[B14] De Swert KO Bracke KR Demoor T Brusselle GG Joos GF Role of the tachykinin NK1 receptor in a murine model of cigarette smoke-induced pulmonary inflammation Respir Res 2009 10 37 10.1186/1465-9921-10-37 19445658 PMC2689186

[B15] Bracke KR D’Hulst AI Maes T Moerloose KB Demedts IK Lebecque S Joos GF Brusselle GG Cigarette smoke-induced pulmonary inflammation and emphysema are attenuated in CCR6-deficient mice J Immunol 2006 177 4350 4359 16982869 10.4049/jimmunol.177.7.4350

[B16] Shan M Yuan X Song LZ Roberts L Zarinkamar N Seryshev A Zhang Y Hilsenbeck S Chang SH Dong C Cigarette smoke induction of osteopontin (SPP1) mediates T(H)17 inflammation in human and experimental emphysema Sci Transl Med 2012 4 117 119 10.1126/scitranslmed.3003041 PMC3956594 22261033

